# Mechanistic insights into the intrinsic drought resilience of *Coffea* spp. and its reinforcement by elevated air CO_2_: from water relations to photoprotection and lipid–sugar dynamics

**DOI:** 10.3389/fpls.2026.1878474

**Published:** 2026-07-30

**Authors:** José N. Semedo, Isabel P. Pais, António E. Leitão, Ana P. Rodrigues, Isabel Marques, Maria J. Silva, Fábio L. Partelli, Fernando C. Lidon, Fábio M. DaMatta, Ana I. Ribeiro-Barros, José C. Ramalho

**Affiliations:** 1 Unidade de Investigação em Biotecnologia e Recursos Genéticos, Instituto Nacional de Investigação Agrária e Veterinária, I.P. (INIAV), Oeiras, Portugal; 2 Plant Stress Ecophysiology and Biochemistry Laboratory, Forest Research Centre (CEF), Associate Laboratory TERRA, School of Agriculture, University of Lisbon (ISA/ULisboa), Oeiras, Portugal; 3 Plant Stress Ecophysiology and Biochemistry Laboratory, Forest Research Centre (CEF), Associate Laboratory TERRA, School of Agriculture, University of Lisbon (ISA/ULisboa), Lisboa, Portugal; 4 Centro Univ. Norte do Espírito Santo (CEUNES), Dept. Ciências Agrárias e Biológicas (DCAB), Univ. Federal Espírito Santo (UFES), São Mateus, ES, Brazil; 5 Department of Earth Sciences, Faculty of Sciences and Technology, NOVA University of Lisbon, Caparica, Portugal; 6 Departamento Biologia Vegetal, Univ. Federal Viçosa (UFV), Viçosa, MG, Brazil

**Keywords:** climate change, coffee tree, drought, elevated CO_2_ stress mitigation, membrane lipids, photoprotection, sugars

## Abstract

Drought is a critical climatic constraint to coffee, but its interaction with enhanced atmospheric [CO_2_] is highly relevant to the crop’s sustainability under climate-change scenarios. We explored such interaction through the physiological and biochemical responses of 7-year-old potted plants of two genotypes: *Coffea canephora* cv. Conilon Clone 153 (CL153) and *C. arabica* cv. Icatu, grown under well-watered (WW) conditions at ambient [CO_2_] (aCO_2_, 380 µL L^-1^) or elevated [CO_2_] (eCO_2_, 700 µL L^-1^). Plants were gradually exposed to moderate (MWD) and severe (SWD) water deficits, reaching predawn water potentials (Ψ_pd_) between -1.6 and -2.2 MPa (MWD) or ≤ -3.7 MPa (SWD). Under aCO_2_, both genotypes showed full resilience to MWD. Moreover, Icatu exhibited tolerance to SWD regardless of [CO_2_], showing stable chronic photoinhibition (PI_Chr_), chlorophyll (Chl) content, and moderate osmotic adjustment from MWD to SWD (mostly related to mannitol buildup). It also showed a broader coordinated response involving enhanced photoprotection (e.g., xanthophylls), the reconfiguration of photosystems (Chl (*a*/*b*) decline), and the lipid profile of chloroplast membranes (fatty-acid composition and degree of unsaturation). The latter involved both quantitative (*de novo* synthesis) and qualitative changes (unsaturation shift, mostly due to an increase in C18:3 and a decline in C16:0), along with an increased C16:1*c+t*. Notably, eCO_2_ attenuated the declines in Ψ_pd_ and cell turgor under MWD particularly in Icatu, thus preserving growth. As for CL153, several adverse impacts were found in SWD under aCO_2_ (PI_Chr_, Chl loss, and membrane leakage), which were counteracted by eCO_2_, highlighting its key role in the acquired resilience of this genotype. Collectively, these findings identify reliable traits underpinning the drought resilience of climate-resilient coffee cultivars.

## 1 Introduction

Drought stands out as a major bottleneck to agricultural productivity, quality, and global food security, disrupting morphological, physiological, and biochemical processes, adversely affecting growth and development, nutrient uptake, C-assimilation and partitioning (Chaves et al., 2003; Wang et al., 2003; Li et al., 2009; Lamaoui et al., 2018), and drastically reducing yields of major staple food crops by 40% to 50% (Qiao et al., 2024). Moderate-to-severe water deficit, even of short duration, can impair multiple components of the photosynthetic apparatus to variable extents. Stomata closure is an early response to an increasing water deficit, reducing water loss by transpiration, but also limiting latent heat loss (thereby increasing leaf temperature), and reducing C-assimilation by limiting CO_2_ diffusion into the leaf (Menezes-Silva et al., 2017; DaMatta et al., 2025). Under progressive drought severity, non-stomatal impairments become increasingly important at photochemical and biochemical levels (Chaves et al., 2003; Matos et al., 2010; Ramalho et al., 2014; Fahad et al., 2017). In addition, harsher conditions will be imposed under the simultaneous occurrence of other limiting conditions, particularly heat and high irradiance, promoting to photoinhibition (Powles, 1984) and broadly constraining cellular processes. Under such conditions, a decline in energy use through photochemistry can secondarily boost the production of highly reactive oxygen species (ROS) and chlorophyll (Chl). This can exacerbate cellular oxidative stress in key structures, namely, the photosynthetic apparatus, by affecting photosystem (PS) proteins and promoting membrane lipoperoxidation (Logan, 2005; Osakabe et al., 2014; Dumanović et al., 2021), resulting in chronic photoinhibition of photosynthesis and reduced plant productivity (Chaves et al., 2008).

Metabolic reprogramming has a key role in plant acclimation to climatic extremes (Zinta et al., 2014; 2018; Marques et al., 2024). Under water deficit conditions, plants can activate the synthesis and accumulation of compatible solutes to preserve osmotic homeostasis and cell turgor, namely, of non-structural sugars [e.g., sucrose, glucose, fructose, and raffinose family oligosaccharides, (RFOs)], and sugar alcohols (e.g., sorbitol and mannitol). They act as osmoprotectants that stabilize membranes and protein conformation by promoting hydration around them (Rejšková et al., 2007; Lahuta and Górecki, 2011; Saddhe et al., 2021), as well as ROS scavengers, especially sugar alcohols (Shen et al., 1997; Chan et al., 2011; Keunen et al., 2013). The drought acclimation response also includes the synthesis of other protective molecules with energy dissipation and antioxidant roles (Logan, 2005; Dumont and Rivoal, 2019). Among these, photoprotective pigments (e.g., zeaxanthin, lutein, and β-carotene) control ROS formation and scavenging, and promote the thermal dissipation of excess radiant energy, acting at the level of antenna complexes and PS reaction centers (Giossi et al., 2020; Dumanović et al., 2021; Demmig-Adams et al., 2022). Antioxidant defenses further include enzymes (e.g., of the Haliwell–Asada cycle and catalase), and other non-enzymatic molecules (e.g., ascorbate, glutathione, HSP70, aquaporins, and dehydrins) (Asada, 2006; Choudhury et al., 2017; Ávila et al., 2020b; Dumanović et al., 2021). Complementarily, sugars are also associated with membrane stabilization and ROS scavenging, being part of stress perception mechanisms and crosstalk in abiotic stress pathways, acting in signaling, osmotic adjustment, and osmotic homeostasis to maintain cell turgor and protein conformation under water deficit (Chaves et al., 2003; Rejšková et al., 2007; Keunen et al., 2013; Saddhe et al., 2021). Collectively, these mechanisms safeguard the structural integrity of chloroplast and cellular membranes and ensure the proper functioning of photosynthetic and metabolic proteins.

Among the most important stress impacts are those imposed on cell membranes, often involving the lipoperoxidation of polyunsaturated fatty acids (PUFAs) due to direct ROS action (Higashi et al., 2015; Niu and Xiang, 2018), and the action of phospholipases and galactolipases that are stimulated under stress conditions (Sahsah et al., 1998). The lipid matrix of chloroplast membranes is mostly composed of polar lipids (phospholipids and galactolipids), with their functional performance strongly dependent on their fluidity, which in turn is closely related to the predominant PUFAs (Xin and Browse, 2000). To keep fluidity, integrity, and function, membranes undergo dynamic lipid remodeling, particularly at the chloroplast level, through *de novo* synthesis and/or modification of pre-existing FAs. This is a vital feature and indicator of stress acclimation, particularly in response to heat (Higashi et al., 2015; Niu and Xiang, 2018), high irradiance (Ramalho et al., 1998), and drought (Perlikowski et al., 2016; Zhang et al., 2018; Liu et al., 2019).

The *Coffea* genus comprises at least 131 species (Royal Botanic Gardens, 2025), but the coffee trade relies on *Coffea arabica* L. (Arabica coffee), and *Coffea canephora* Pierre ex A. Froehner (Robusta coffee), currently contributing to approximately 57% and 43% of worldwide coffee bean yield, respectively (DaMatta et al., 2025). The coffee value chain is a major source of income for approximately 25 million smallholder farmers in more than 80 producing countries, almost exclusively from the tropical belt (DaMatta et al., 2019; Pham et al., 2019), with a global involvement of 60 to 125 million people (Sachs et al., 2019).

Forecasts point to the likelihood that coffee crop performance will be strongly affected by climate change (CC), with implications from farmers to consumers. Impacts will be mostly related to warming and increased drought frequency and severity, causing losses in yield, biodiversity of wild coffee species (Davis et al., 2019; Martins et al., 2025), and bean sensory attributes (Ahmed et al., 2021; Ramalho et al., 2018b). In addition, future climate conditions will reduce adequate crop areas and increase pest and disease incidence (Magrach and Ghazoul, 2015; Pham et al., 2019). Still, enhanced atmospheric [CO_2_] (eCO_2_) (negatively associated with global warming) has been found to boost photosynthetic performance, plant vigor (Ramalho et al., 2013b; Ghini et al., 2015; Semedo et al., 2018), investment in reproductive structures (Rakočević et al., 2020), and yield under adequate water supply (Verhage et al., 2017; DaMatta et al., 2019). eCO_2_ directly stimulates photosynthesis and reduces photorespiration, often increasing net photosynthesis (P_n_) by 50% in woody species (Ainsworth and Rogers, 2007), including in coffee (Ramalho et al., 2013b; Ghini et al., 2015; Ávila et al., 2020a). This its due to its C3 photosynthesis and also because eCO_2_ largely overcomes this plant’s high diffusional constraints of CO_2_ flow at the stomata and mesophyll levels. In fact, the increase in global coffee yield in the last decades would have resulted from the adoption of more efficient management practices to mitigate such impacts (e.g., agroforestry), the use of elite genotypes, and also the potential contribution of the progressive increase in air [CO_2_] (DaMatta et al., 2025). Yet, the eCO_2_ beneficial effect on C-assimilation, growth, and plant biomass production may be greatly compromised by single or combined stresses, their severity, and duration (Abo Gamar et al., 2019; Birami et al., 2020; Tausz-Posch et al., 2020). In addition, eCO_2_ might interfere with the plant acclimation ability in a species/genotype-dependent basis. Studies suggest that eCO_2_ may not alleviate the negative impacts of drought stress on soybean photosynthesis, although it can play an important role in drought stress responses (Li et al., 2020), while in other species (e.g., *Caragana korshinskii*), it may alleviate the negative impacts of high temperature and salinity on phytohormones, photosynthesis, and redox reactions, namely, through the control of ROS generation and extent of lipoperoxidation (Yan et al., 2025). Still, despite the mitigation of heat and drought impacts at the physiological level (Abdelhakim et al., 2022), no benefit was found on wheat yield (Abdelhakim et al., 2022; Chang et al., 2023). In *Coffea* spp., eCO_2_ was found to mitigate the stress impacts promoted by heat (Martins et al., 2016; Rodrigues et al., 2016; Scotti-Campos et al., 2019), also by drought, by prompting a number of common mechanisms particularly associated with the protection of the photosynthetic apparatus. In fact, the enhanced drought resilience associated with eCO_2_ aligned with the ability to trigger a coordinated set of metabolic defense responses, including amplified levels of photoprotective and antioxidative components (e.g., enzymes), and other molecules (e.g., thylakoid electron carriers involved in cyclic electron flow, amino acids, and aquaporins) (Avila et al., 2020a; Avila et al., 2020b; Rodrigues et al., 2021; Semedo et al., 2021; Marques et al., 2022b, 2023; Martins et al., 2025). Together, such mechanisms promote photochemical energy use and/or dissipation (both limiting the formation of ROS, and boost other protective mechanisms related to ROS scavenging, thus contributing to coffee plant acclimation/resilience and to the sustainability of this crop under future CC conditions.

Considering that the preservation of water balance, cellular membrane integrity, and overall metabolic functioning are crucial for maintaining plant performance under changing climate conditions, we expand current knowledge regarding the complementary mechanisms underpinning an intrinsic drought resilience in coffee and its modulation by eCO_2_, targeting key physiological and biochemical traits mostly associated with the photosynthetic machinery. To this end, an integrated analysis of plant water relations, PSII function, and dynamic responses of photoprotection, non-structural carbohydrates, membrane selectivity, and chloroplast membrane lipid profile was performed in two cropped genotypes representing the two main coffee-producing species. Such insights are vital for sustaining coffee productivity under future CC scenarios, in which water scarcity events are expected to be increasingly frequent and more severe, together with rising atmospheric [CO_2_].

## 2 Materials and methods

### 2.1 Plant material and growth conditions

A total of 36 plants representing two Brazilian cultivated genotypes (18 of each), belonging to the two main coffee-producing species were used: *Coffea canephora* Pierre ex A. Froehner cv. Conilon Clone 153 (CL153) and *C. arabica* L. cv. Icatu Vermelho (an introgressed variety resulting from a cross between *C. canephora* and *C. arabica* cv. Bourbon Vermelho, further crossed with *C. arabica* cv. Mundo Novo). The plants were grown from the seedling stage for 7 years, in 80-L pots, distributed to two walk-in growth chambers (EHHF 10000, ARALAB, Portugal), under controlled temperature (25 °C/20 °C, day/night, ± 1 °C), irradiance (approximately 750 μmol m^-2^ s^-1^ at the upper part of the plant), relative humidity (70% ± 2%), photoperiod (12 h), and either at ambient (aCO_2_, 380 ± 5 μL L^-1^) or elevated (eCO_2_, 700 ± 5 μL L^-1^) air [CO_2_], the latter being projected to be reached during the second half of the 21st century (IPCC, 2014). Potted plants were grown in a substrate consisting of a mixture of soil, peat, and sand (3:1:3, v/v/v), with a pH of 6.5, and were maintained under adequate soil moisture by watering the plants every 2 days (until water treatment), and without restrictions in nutrients (see Ramalho et al., 2013a; Ramalho et al., 2013b), or root development (as visually examined at the end of the experiment after removing the plants from their pots). To minimize any possible “growth chamber effect”, both chambers were regularly calibrated by the manufacturer to maintain identical environmental conditions (air humidity, temperature, light intensity, and quality) (with the exception of air [CO_2_]), and plants were frequently swapped between chambers (Semedo et al., 2021).

Sample collection and evaluations were performed on newly matured leaves from the upper third part (well illuminated), in photosynthetic steady-state conditions after approximately 2 h of illumination, except for predawn water potential and dark-adapted fluorescence measurements. For biochemical evaluations, leaf samples were immediately frozen in liquid nitrogen and stored at −80 °C, being finely grounded in liquid N_2_ prior to analysis. Leaf tissue extractions were performed using an ice-cold mortar and pestle and cold homogenizing solutions.

### 2.2 Water deficit imposition

Water deficit was imposed as explained in detail in Semedo et al. (2021). Briefly, within each growth chamber, which differed only in air [CO_2_], plants were divided into three groups of six plants: (1) well-watered (WW) throughout the experiment, with leaf predawn water potential (Ψ_pd_) kept at values > -0.35 MPa), (2) moderate water deficit (MWD) to reach Ψ_pd_ between –1.5 and –2.5 MPa, and (3) severe water deficit (SWD) to reach Ψ_pd_ < –3.5 MPa). For MWD and SWD treatments, water was gradually withheld over 2 weeks, until the target Ψ_pd_ values were reached. Such conditions represented approximately 80% (WW), 25% (MWD) or 10% (SWD) of the maximum pot water availability (Ramalho et al., 2018a). After reaching the target Ψ_pd_ values under MWD or SWD treatments, pot moisture was maintained for an additional 2 weeks by adding adequate amounts of water before sampling and evaluation.

Exceptionally, the Icatu 700-plants under MWD conditions were exposed to complete water withholding during the last 5 days of the initial 4-week period, to further reduce Ψ_pd_ values, which, even so, did not decrease below -0.6 MPa.

### 2.3 Leaf water status

Leaf Ψ_pd_ was measured immediately after leaf excision at the end of the night period, using a pressure chamber (Model 1000, PMS Instrument Co., Albany, OR, USA), with five to six true replicates per treatment.

Leaf relative water content (RWC) was evaluated using samples of 10 leaf discs (0.5 cm^2^ each) punched from similar leaves used for Ψ_pd_ determinations. RWC was calculated as RWC (%) = [(FW – DW)/(TW – DW)] × 100, where fresh weight (FW) was determined immediately after cutting the discs. Turgid weight (TW) was determined after overnight rehydration of the discs in a humid chamber at room temperature (approximately 20 °C), and dry weight (DW) was obtained after drying the discs at 80 °C for 24 h.

The Ψ_π_ determination followed Melkonian et al. (2004), using samples of 15 leaf discs (0.5 cm^2^ each) from the leaves used for RWC evaluation. The discs were punched, immediately fast-frozen in liquid N_2_, and stored at -80 °C. For evaluations, each sample was placed in the steel cups of a thermocouple psychrometer (SC-10A, Decagon Devices Inc., Pullman, WA, USA) and allowed to equilibrate for 2 h before the readings. The psychrometer output (µV) was converted to osmotic potential (MPa) using a calibration curve generated with eight known KCl solutions (between 0 and 1 M), spanning the expected Ψ_π_ range. Such Ψ_π_ values were then corrected to full turgor using the RWC of the same sample [Ψ_π100_ = Ψ_π_ × (RWC/100)] (Mendes et al., 2001).

Turgor pressure (Ψ_p_) was calculated using the equation, Ψ_pd_ = Ψ_π_ + Ψ_p_ (Melkonian et al., 2004), where Ψ_π_ represents the measured osmotic potential prior to correction to full turgor, and Ψ_pd_ was assumed to be a proxy for maximum leaf water potential, with values > 0 indicating conditions that allow cell expansion.

### 2.4 Photosystem II performance

To assess the impact of water deficit on the performance of photosystem II (PSII), chlorophyll *a* fluorescence was measured with a PAM-2000 system (H. Walz, Effeltrich, Germany), as detailed for *Coffea* spp (Rodrigues et al., 2016). The maximum (F_v_/F_m_) and actual (F_v_’/F_m_’) photochemical efficiencies of PSII were evaluated in overnight dark-adapted leaves, and under photosynthetic steady-state conditions, respectively (Semedo et al., 2021). Subsequently, they were used for the calculation of the photoinhibition indexes: (i) dynamic photoinhibition (PI_Dyn_), representing the decline of F_v_/F_m_ that is fully reversible at night, being measured as the percentage reduction of F_v_’/F_m_’ compared with F_v_/F_m_ for each treatment, compared with to the maximum F_v_/F_m_ value recorded in the entire experiment; (ii) chronic photoinhibition (PI_Chr_), representing the percentage reduction of F_v_/F_m_ in each treatment in comparison with the maximum F_v_/F_m_ recorded in the entire experiment; and (iii) total photoinhibition, representing the sum of the previously calculated indexes (PI_Total_ = PI_Chr_ + PI_Dyn_) (Werner et al., 2002).

### 2.5 Chlorophyll content

Total chlorophyll (Chl) content was obtained from frozen samples of three 0.5 cm^2^ leaf discs, from four to six plants per treatment, excised after approximately 2 h of leaf exposure to diurnal illumination, flash frozen in liquid nitrogen, and stored at -80 °C until analysis. Each sample was then homogenized in 1.5 mL of 90% aqueous acetone (v/v), centrifuged (10, 000 × *g*, 10 min, 4 °C), and filtered (nylon filter, 13 mm, 0.45 µm). An aliquot from the clear supernatant was then diluted to 80% acetone, and chlorophyll concentrations were determined spectrophotometrically according to the coefficients of Lichtenthaler (1987) for 80% acetone.

### 2.6 Photoprotective pigments evaluation

From the filtered clear supernatant obtained from chlorophyll extraction in 90% acetone (see above), a 50-µL aliquot was used for HPLC analysis, on a Spherisorb ODS-2 (250 x 4.6 mm, 5 µm) reverse-phase C18 column, with guard columns of the same material, as described for coffee leaves (Vinci et al., 2022). Detection was performed at 440 nm using an HPLC system (Beckman, System Gold, Tulsa, USA) coupled with a diode array detector (Model 168; Beckman). Standards of each pigment were used for identification and quantification. The de-epoxidation state (DEPS) involving the xanthophyll cycle components, violaxanthin (Viol), antheraxanthin (Ant), and zeaxanthin (Zea), was calculated according to Schindler et al. (1994) as (DEPS = [(Zea+0.5 Ant)/(Viol+Ant+Zea)]). The presented values for total carotenoid content (Tot Car) represent the sum of individual carotenoids obtained through HPLC analysis.

### 2.7 Non-structural carbohydrates quantification

Sugars were determined in powdered frozen material (approximately 150 mg FW), based on Damesin and Lelarge (2003), and processed as described for coffee leaf material (Ramalho et al., 2013b). For HPLC analysis, a 20-µL aliquot of each sample solution was injected into a system equipped with a refractive index detector (Model 2414, Waters, USA), and a Sugar-Pak I column (300 × 6.5 mm, Waters), maintained at 90 °C, with H_2_O (containing 50-mg EDTA-Ca L^-1^ H_2_O) as the eluent, with a flow rate of 0.5 mL min^-1^. To resolve potential non-pure peaks of this separation, another 20-µL aliquot of each sample was injected through a DionexCarboPac PA1 analytical column (4 × 250 mm, Thermo Scientific, Waltham, MA, USA) coupled to a DionexCarboPac PA1 Guard (4 × 50 mm) at 20 °C. Ultrapure water and 300-mM NaOH were used as eluents (water: 0–50 min; NaOH: 50−65 min; and water: 65−80 min for re-equilibration), at a flow rate of 1 mL min^-1^. Standard curves were used to quantify each sugar.

Starch quantification was accomplished following Stitt et al. (1978) with some modifications described for coffee leaf material (Ramalho et al., 2013b). Starch was then enzymatically hydrolyzed to glucose and quantified by measuring absorbance at 340 nm.

Total carbohydrates represent the sum of sugars (soluble) and starch (insoluble).

### 2.8 Membrane permeability assessment

Leaf cellular membrane permeability/integrity was assessed as previously described for coffee plants (Scotti-Campos et al., 2019). Briefly, composite samples of 10 leaf discs (0.5 cm^2^ each) were rinsed three times with deionized water and placed to float in 15-mL deionized water (24 h, 20 °C). The electrolyte leakage of each sample was then assessed by measuring water conductivity using a Crison GLP31 (Crison Instruments, S.A., Barcelona, Spain). The samples were then exposed to 90 °C for 2 h for complete membrane permeabilization, followed by cooling to 20 °C, after which total conductivity was measured. Membrane leakage of each sample was expressed as the percentage of its total conductivity.

### 2.9 Fatty acid profile from chloroplast membranes

The extraction and further processing of leaf material to obtain the fatty acid (FA) profile from enriched chloroplast membrane fractions were performed using 4-g FW of leaf tissues, as previously optimized and described in detail for coffee leaves (Partelli et al., 2011; Scotti-Campos et al., 2019). Briefly, freshly cut leaf material was immediately homogenized in 25 mL of cold 50-mM MES buffer (pH 6.4), containing 0.4-M D-sorbitol, 10-mM NaCl, 5-mM MgCl_2_, 2-mM EDTA, 1-mM MnCl_2_, 0.4% (w/v) BSA, and 2-mM Na-ascorbate. Subsequently, the homogenate was filtered with eight layers of cheesecloth and centrifuged at 3, 000 × *g* for 5 min at 4 °C. The obtained chloroplast pellet was used for lipid extraction by mixing it with 9 mL of a chloroform/methanol/water (1/1/1, v/v/v) solution, followed by centrifugation at 4, 500 × *g* for 10 min at 4 °C. The lower chloroform phase was evaporated to dryness under N_2_ flux, and the lipid residue was resuspended in 1.5 mL of an ethanol:toluene (1:4) mixture, to be used for subsequent analyses. For FA analysis, a 50-μL aliquot of the previous lipid resuspension was saponified and methylated with BF3. To quantify FAs, heptadecanoic acid (C17:0) was added to each sample as an internal standard. Total lipid extraction followed Allen et al. (1966), and lipid methylation was carried out according to Metcalfe et al. (1966).

FA methyl esters (FAME) were analyzed by GC-FID (Varian, CP-3380, USA), with a DB-Wax capillary column (J & W Scientific, USA, 0.25-mm i.d. × 30 m, 0.25 μm). Column temperature was programmed to rise from 80 °C to 200 °C at 12 °C min^-1^, after 2 min at the initial temperature. Injector and detector temperatures were 200 °C and 250 °C, respectively. The carrier gas was hydrogen with a flow rate of 1 mL min^-1^, at a split ratio of 1:50. Individual FAs were identified by comparison with a standard mixture (FAME Mix, Restek, USA). Total FAs (TFA) denote the sum of individual FAs. The degree of TFA unsaturation was calculated as the double bond index (DBI = [(%monoenes + 2 × %dienes + 3 × %trienes)/%saturated FAs]), following Mazliak (1983).

Possible lipid contamination of the chloroplast membrane-enriched fractions with other cellular membranes can be considered negligible, as previously shown for the same method and plant species (Partelli et al., 2011; Scotti-Campos et al., 2019).

### 2.10 Experimental design and statistical analysis

The experimental design followed Semedo et al. (2021). Plants of each genotype were exposed to six treatments, from a 2 × 3 factorial (two [CO_2_], aCO_2_ or eCO_2_ and three levels of water availability, WW, MWD, or SWD), following a completely randomized design, with six plants (one per pot) per treatment. A two-way ANOVA was used to assess the differences between air [CO_2_] (aCO_2_ or eCO_2_), between water availability (WW, MWD, or SWD), and their interaction, separately for each genotype. Subsequently, *a posteriori* Tukey’s honestly significant difference (HSD) test for mean comparisons was performed. Data were analyzed using STATISTICA v7.0 (StatSoft, Hamburg, Germany). A 95% confidence level was adopted for all tests.

## 3 Results

### 3.1 Characterization of leaf water status

Leaf water potential at predawn (Ψ_pd_) showed high and close values (−0.30 to −0.36 MPa) in well-watered plants (WW) regardless of air [CO_2_] and genotype (**Table 1**). The gradual water deficit implementation progressively declined Ψ_pd_ values to moderate (MWD: −1.6 to −2.2 MPa, except in Icatu 700-plants, −0.62 MPa) and highly severe (SWD: −3.7 and −4.5 MPa) drought, usually similar among genotypes within the 2-week period of reduced irrigation. Still, eCO_2_ promoted higher Ψ_pd_ values under MWD in both genotypes (significant in Icatu), compared with aCO_2_ plants. Notably, the higher Ψ_pd_ value in Icatu-MWD-eCO_2_ plants (-0.62 MPa) was stable even under a total irrigation suspension for 5 days prior to sample collection.

**Table 1:** Characterization of plant water relations, through the evaluation of leaf water potential (Ψ_pd_), osmotic potential corrected to full turgor conditions (Ψ_π100_), and turgor pressure (Ψ_p_), all in samples collected at predawn, in *Coffea canephora* cv. Conilon Clone 153 (CL153) and *Coffea arabica* cv.

Genotype	[CO_2_]	Water treatment	Ψ_pd_				Ψ_π100_				Ψ_p_			
	(μL L^−1^)		(MPa)				(MPa)				(MPa)			
CL153	380	WW	-0.310	±	0.031	aA	-1.83	±	0.33	abA	1.64	±	0.43	aA
		MWD	-2.16	±	0.19	bA	-1.52	±	0.10	aA	-0.250	±	0.027	bB
		SWD	-3.85	±	0.44	cA	-2.21	±	0.14	bA	-0.281	±	0.295	bA
	700	WW	-0.363	±	0.043	aA	-1.71	±	0.15	aA	1.43	±	0.09	aA
		MWD	-1.59	±	0.25	bA	-1.81	±	0.09	aA	0.311	±	0.242	bA
		SWD	-3.71	±	0.51	cA	-1.77	±	0.14	aB	-0.782	±	0.48	cA
Icatu	380	WW	-0.340	±	0.020	aA	-1.92	±	0.11	abA	1.92	±	0.10	aA
		MWD	-1.97	±	0.32	bB	-1.51	±	0.24	aA	-0.602	±	0.348	bB
		SWD	-3.69	±	0.19	cA	-2.08	±	0.20	bA	-0.322	±	0.20	bA
	700	WW	-0.363	±	0.052	aA	-1.92	±	0.14	abA	1.76	±	0.25	aA
		MWD	-0.618	±	0.073	aA	-1.76	±	0.08	aA	1.29	±	0.09	aA
		SWD	-4.56	±	0.34	bA	-2.21	±	0.23	bA	-0.746	±	0.160	bA

Icatu, grown under ambient CO_2_ conditions (380 µL L^−1^, aCO_2_) or elevated CO_2_ (700 µL L^−1^, eCO_2_), and subjected to three water availability levels: well-watered (WW), moderate water deficit (MWD), and severe water deficit (SWD).

For each parameter, different letters after the mean values ± SE (n = 5–6) express significant differences between water availability conditions at the same [CO_2_] (a, b, c) or between the two [CO_2_] under the same water availability condition (A, B), always separately for each genotype.

Predawn leaf osmotic potential corrected to full turgor (Ψ_π100_) in WW plants was similar among [CO_2_] and genotypes. Ψ_π100_ was not significantly altered by the imposition of MWD and SWD, compared with WW plants, although the lowest absolute values were usually found in SWD plants.

With WW conditions, both genotypes exhibited high positive leaf Ψ_p_, which declined progressively with increasing water deficit, in parallel with reductions in Ψ_pd_. However, with MWD the Ψ_p_ became negative under aCO_2_, whereas it remained positive under eCO_2_ in both genotypes, particularly in Icatu, indicating that cell expansion was still physiologically feasible only under eCO_2_ during moderate drought.

Under SWD, Ψ_p_ declined further, reaching values below zero regardless of air [CO_2_] and genotype. Ψ_p_ was estimated indirectly as the difference between Ψ_pd_ and Ψ_π_, which were determined using independent methods. This approach could generate negative Ψ_p_ estimates, although these do not imply the existence of negative turgor pressure, which is physiologically impossible in living leaf cells, but rather reflect the limitations of indirectly deriving Ψ_p_ from independently measured components. Therefore, Ψ_p_ estimates should be interpreted qualitatively, with positive values indicating the presence of turgor, whereas values ≤ 0 reflect effective turgor loss.

### 3.2 Photosystem II photoinhibition status

**Figure 1:**
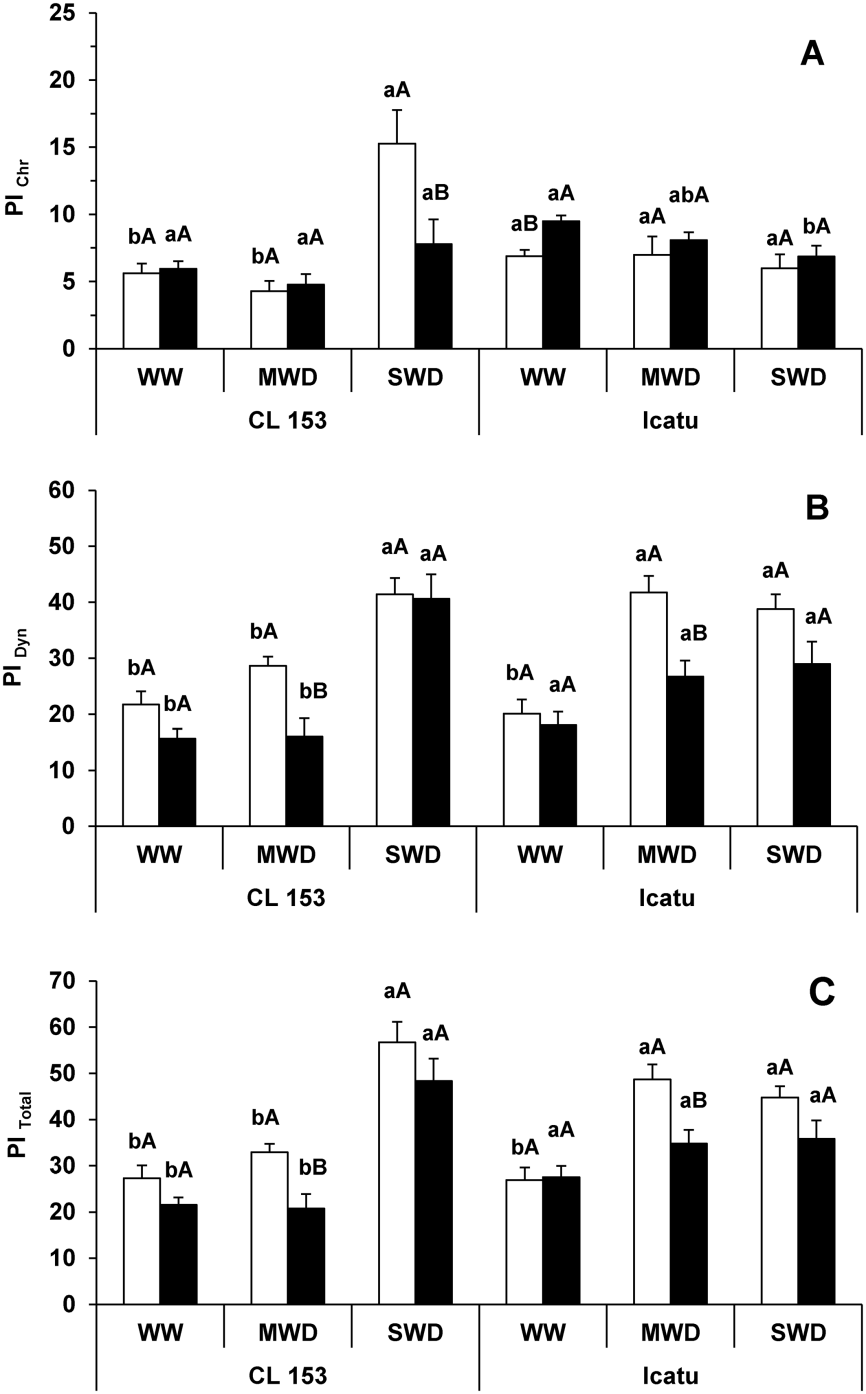
Photochemical performance of photosystem II, assessed through the leaf fluorescence parameters related to the photoinhibition status: **(A)** PI_Chr_ (chronic photoinhibition), **(B)** PI_Dyn_ (dynamic photoinhibition), and **(C)** PI_Total_ (total photoinhibition), in *Coffea canephora* cv. Conilon Clone 153 (CL153) and *C. arabica* cv. Icatu. Plants were grown under ambient CO_2_ conditions (380 µL L^-1^, aCO_2_, white bar) or elevated CO_2_ (700 µL L^-1^, eCO_2_, black bar), and subjected to three water availability levels: well-watered (WW), moderate water deficit (MWD), and severe water deficit (SWD). For each parameter, different letters after the mean values ± SE (n = 5-6) express significant differences between water availability conditions at the same [CO_2_] **(a, b)** or between the two [CO_2_] under the same water availability condition **(A, B)**, always separately for each genotype.

Chronic photoinhibition (PI_Chr_) represented as much as 27% and 16% of PI_Total_ in CL153-SWD plants under aCO_2_ and eCO_2_, respectively (**Figure 1A**), while the corresponding values in Icatu-SWD plants were 13% and 19%, respectively, under aCO_2_ and eCO_2_ []. Notably, dynamic PSII photoinhibition (PI_Dyn_) consistently accounted for the majority of total photoinhibition (PI_Total_) (**Figures 1B, C**).

In CL153-aCO_2_ plants, the PI_Chr_, PI_Dyn_, and PI_Total_ values did not change with MWD, but greatly increased by 172%, 91%, and 107%, respectively, under SWD. Notably, Icatu-aCO_2_ plants showed stable PI_Chr_ values in MWD and SWD, but PI_Dyn_ doubled (with a parallel increase of approximately 80% in PI_Total_) under single MWD and SWD exposure. In general, eCO_2_ did not impact these parameters in WW plants (except for PI_Chr_ in Icatu). Furthermore, under eCO_2_, PI_Chr_ was not altered in MWD and SWD in both genotypes, with a significant reduction in CL153-SWD plants when compared with their aCO_2_ values. Notably, eCO_2_ reduced PI_Dyn_ (and PI_Total_), during drought (significantly in MWD) in both genotypes.

### 3.3 Photosynthetic pigments dynamic

A single drought event decreased the total chlorophyll content [Chl (*a*+*b*)] in CL153 plants under MWD (35%) and SWD (25%), whereas no effect was observed in Icatu (**Table 2**). In contrast, under eCO_2_, Chl (*a*+*b*) remained stable in SWD plants of both genotypes compared with their respective WW plants. The Chl *a*/*b* ratio was not greatly affected by the imposed treatments, but showed a clear tendency to decrease in Icatu, significantly only in Icatu-SWD plants under aCO_2_.

**Table 2:** Leaf photosynthetic pigments, including total chlorophyll (*a*+*b*), chlorophyll *a*/*b* ratio, individual xanthophylls and carotenes, and total carotenoids, Tot Car, assessed in *Coffea canephora* cv. Conilon Clone 153 (CL153) and *C. arabica* cv. Icatu.

Genotype		[CO2]	Water treatment	Chl (a+b)						Chl (a/b)						Neoxanthin					Zeaxanthin						Viol+Ant+Zea				DEPS*				
		(mL L^-1^)		(mg g^-1^ DW)						(g g^-1^)						(mg g^-1^ DW)					(mg g^-1^ DW)						(mg g^-1^ DW)								
CL153		380	WW	10.23		±	0.42		aA	3.19		±	0.06		aA	0.243		±	0.01	aA	0.047		±	0.006		bA	0.347	±	0.011	abA	0.246	±	0.042	bA
			MWD	6.58		±	0.93		bA	3.38		±	0.09		aA	0.146		±	0.02	bA	0.079		±	0.014		bA	0.257	±	0.024	bA	0.424	±	0.074	abA
			SWD	7.63		±	0.57		bA	3.22		±	0.1		aA	0.179		±	0.015	abB	0.235		±	0.047		aA	0.394	±	0.038	aA	0.665	±	0.068	aA
		700	WW	10.51		±	0.86		aA	3.29		±	0.06		aA	0.243		±	0.018	aA	0.083		±	0.016		bA	0.347	±	0.022	aA	0.252	±	0.031	bA
			MWD	8.22		±	0.33		aA	3.21		±	0.09		aA	0.191		±	0.011	aA	0.125		±	0.036		bA	0.345	±	0.041	aA	0.304	±	0.041	bA
			SWD	10.59		±	1.27		aA	3.13		±	0.04		aA	0.255		±	0.029	aA	0.299		±	0.056		aA	0.463	±	0.025	aA	0.612	±	0.084	aA
Icatu		380	WW	9.85		±	0.43		aA	3.14		±	0.08		aA	0.214		±	0.007	bA	0.048		±	0.006		cA	0.36	±	0.013	bA	0.181	±	0.018	bA
			MWD	10.28		±	0.56		aA	2.99		±	0.05		abA	0.184		±	0.025	bA	0.216		±	0.045		bA	0.397	±	0.039	bA	0.619	±	0.058	aA
			SWD	11.2		±	0.63		aA	2.82		±	0.05		bA	0.283		±	0.017	aB	0.403		±	0.034		aA	0.552	±	0.033	aA	0.735	±	0.029	aA
		700	WW	10.51		±	0.68		aA	3.08		±	0.06		aA	0.218		±	0.01	bA	0.044		±	0.008		bA	0.308	±	0.01	bA	0.183	±	0.029	bA
			MWD	7.37		±	0.51		bB	2.97		±	0.06		aA	0.183		±	0.016	bA	0.123		±	0.059		bA	0.378	±	0.074	bA	0.288	±	0.09	bB
			SWD	12.27		±	0.58		aA	2.87		±	0.05		aA	0.348		±	0.012	aA	0.309		±	0.044		aA	0.616	±	0.029	aA	0.575		0.05	aB
(Viol+Ant+Zea)/Tot Car				Lutein						a-Carotene						b-Carotene					(a+b) Carotene						(a/b) Carotene				Tot Car				
(mg g^-1^ DW)				(mg g^-1^ DW)						(mg g^-1^ DW)						(mg g-^-1^ DW)					(mg g^-1^ DW)						(g g^-1^)				(mg g^-1^ DW)				
0.199	±	0.005	bA	0.785	±	0.033		aA		0.178	±	0.012		aA		0.19	±	0.016		aA	0.368	±	0.024		aA		1.15	±	0.12	bA	1.69	±	0.04		aA
0.24	±	0.01	abA	0.481	±	0.063		bA		0.176	±	0.02		aA		0.114	±	0.003		abA	0.29	±	0.018		abA		1.19	±	0.12	abA	1.4	±	0.06		aA
0.275	±	0.018	aA	0.631	±	0.045		abA		0.157	±	0.013		aA		0.098	±	0.004		bA	0.254	±	0.017		bA		1.58	±	0.09	aA	1.54	±	0.09		aB
0.212	±	0.006	bA	0.746	±	0.043		aA		0.205	±	0.035		aA		0.224	±	0.021		aA	0.429	±	0.052		aA		0.894	±	0.095	bA	1.64	±	0.09		abA
0.235	±	0.021	abA	0.619	±	0.019		aA		0.132	±	0.009		aA		0.153	±	0.008		bA	0.284	±	0.012		bA		0.891	±	0.081	bA	1.44	±	0.06		bA
0.281	±	0.02	aA	0.773	±	0.068		aA		0.214	±	0.03		aA		0.134	±	0.009		bA	0.348	±	0.038		abA		1.53	±	0.12	aA	1.89	±	0.15		aA
0.221	±	0.005	bA	0.713	±	0.025		bA		0.131	±	0.015		bA		0.206	±	0.009		bA	0.338	±	0.032		aA		0.627	±	0.06	bA	1.64	±	0.04		bA
0.25	±	0.018	abA	0.626	±	0.033		bA		0.092	±	0.007		bA		0.272	±	0.031		aA	0.365	±	0.027		aA		0.391	±	0.069	bA	1.58	±	0.06		bA
0.264	±	0.01	aA	0.928	±	0.055		aA		0.198	±	0.019		aA		0.144	±	0.005		cA	0.342	±	0.021		aA		1.36	±	0.13	aA	2.09	±	0.1		aB
0.209	±	0.003	bA	0.681	±	0.029		bA		0.117	±	0.011		bA		0.2	±	0.009		aA	0.317	±	0.017		aA		0.59	±	0.069	bA	1.49	±	0.04		bA
0.254	±	0.025	abA	0.634	±	0.061		bA		0.077	±	0.003		bA		0.142	±	0.009		bB	0.171	±	0.039		bB		0.554	±	0.032	bA	1.41	±	0.14		bA
0.261	±	0.009	aA	1.033	±	0.042		aA		0.2	±	0.023		aA		0.168	±	0.008		bA	0.368	±	0.023		aA		1.23	±	0.14	aA	2.39	±	0.09		aA

* DEPS = [Zea + (0.5 Ant)/(Viol + Ant + Zea)], where Viol is violaxanthin, Ant is antheraxanthin, and Zea is zeaxanthin.

Plants were grown under ambient CO_2_ conditions (380 µL L^-1^, aCO_2_) or elevated CO_2_ (700 µL L^-1^, eCO_2_), and subjected to well-watered (WW), moderate water deficit (MWD), or severe water deficit (SWD). For each parameter, different letters after the mean values ± SE (n = 4–6) express significant differences between water availability conditions at the same [CO_2_] (a, b, c) or between the two [CO_2_] under the same water availability condition (A, B), always separately for each genotype.

As regards carotenoids, the total content of individual xanthophylls and carotenes (Tot Car) showed no changes under MWD conditions, regardless of genotype or air [CO_2_]. However, in Icatu, the SWD promoted significant increases of 27% (aCO_2_) and 60% (eCO_2_) in Tot Car, the latter resulting in the greater absolute content and increase relative to WW plants. Notably, with eCO_2_ and SWD superimposition, CL153 also showed a significant rise (23%), compared with their SWD-aCO_2_ plants.

Tot Car variations reflected specific modifications of individual carotenoids, influenced by water availability, air [CO_2_], and genotype (**Table 2**). Neoxanthin level was reduced during drought in CL153-aCO_2_, but eCO_2_ cancelled this effect, resulting in a 42% higher concentration in SWD plants grown under eCO_2_ compared with aCO_2_. In contrast, neoxanthin greatly increased in Icatu-SWD plants in both air [CO_2_], but to a greater extent in eCO_2_, resulting in a 23% higher value than under aCO_2_.

The pool of xanthophyll cycle pigments (violaxanthin, Viol; antheraxanthin, Ant; and zeaxanthin, Zea) broadly exhibited a similar trend to that of neoxanthin, although displayed distinct patterns of variation. Among these pigments, the photoprotective/dissipative Zea stood out, with larger and progressive variations as drought severity increased under both [CO_2_] and in both genotypes. Maximum Zea values were found under SWD, with increases of 4.0-fold (aCO_2_) and 2.6-fold (eCO_2_) in CL153, and 7.4-fold (aCO_2_) and 6.0-fold (eCO_2_) in Icatu, compared with those of their WW plants, but without significant differences between CO_2_ treatments in either genotype. This increase in Zea led to a gradual increase of both DEPS and the relative proportion of the (Viol + Ant + Zea) pool relative to Tot Car [(Viol + Ant + Zea)/Tot Car], reaching maximum values under SWD for both genotypes and [CO_2_] treatments.

Interestingly, a tendency for lower dissipation requirements (assessed through DEPS) was usually found under eCO_2_ compared with the corresponding aCO_2_ conditions under MWD and SWD. Notably, in CL153, the reinforced photoprotective potential associated with the Zea increase was largely supported by the pre-existing (Viol + Ant + Zea) pool present under WW, as the abundance of this pool increased only marginally under either [CO_2_] during drought. In contrast, the Zea increase in Icatu was primarily related to a progressive *de novo* synthesis of the (Viol + Ant + Zea) pool. This was particularly evident under SWD where maximum absolute contents were observed due to increases of 53% (aCO_2_) and 100% (eCO_2_), without differences between CO_2_ treatments at any water availability level.

Lutein, the most abundant carotenoid, showed distinct variations between genotypes. Whereas CL153 plants showed no significant differences between WW and SWD conditions regardless of [CO_2_], Icatu plants showed significant rises of 30% (aCO_2_) and 52% (eCO_2_) under SWD, tending to a greater content (11%) under eCO_2_.

Regarding carotenes, under SWD, the α-carotene content was largely stable in CL153, but significantly increased in Icatu (aCO_2_: 51%; eCO_2_: 71%), without differences between [CO_2_] for either genotype. In contrast, β-carotene values declined with increasing drought severity, with low values under SWD in both genotypes and [CO_2_], but a minor decline (16%) was observed in Icatu-eCO_2_ plants, relative to their WW plants. These contrasting α- and β-carotene variations led to a decline in the total (α+β)-carotene of CL153-SWD plants, whereas the values were largely maintained in Icatu-SWD, under both [CO_2_]. Moreover, they resulted in the maintenance of the (α/β)-carotene ratio under MWD, and with strong increases under SWD, in both genotypes and [CO_2_]. Still, it is worth underlining that the declines in (α/β)-carotene ratio had distinct causes resulting from the α- and β-carotene declines in CL153-aCO_2_, a reduction of β-carotene in CL153-eCO_2_, and an increase in α-carotene and a reduction in β-carotene in Icatu.

### 3.4 Non-structural carbohydrates modification

Different changes were observed among sugars depending on genotype, water availability, and air [CO_2_] (**Table 3**). Despite some fluctuations, raffinose was not affected by SWD under aCO_2_. However, eCO_2_ stimulated its content in WW and SWD plants of both genotypes. Trehalose values were strongly altered by drought solely in CL153, with declines of 92% (aCO_2_) and 64% (eCO_2_) under SWD relative to WW controls, but without differences between [CO_2_].

**Table 3:** Leaf non-structural carbohydrates: sugars (mg g^-1^ DW) and starch (mg glucose equivalents g^-1^ DW) assessed in *Coffea canephora* cv. Conilon Clone 153 (CL153) and *C. arabica* cv. Icatu.

Genotype	[CO₂] (µL L⁻¹)	Water treatment	Raffinose				Trehalose				Sucrose				Glucose				Fructose			
**CL 153**	380	WW	0.388	±	0.056	bB	9.97	±	1.17	aA	17.2	±	0.9	aB	0.722	±	0.069	bA	7.09	±	0.43	aA
		MWD	1.1	±	0.17	aA	3	±	0.46	bA	3.87	±	0.54	bB	2.89	±	0.15	aA	9.3	±	0.96	aA
		SWD	0.577	±	0.218	abB	0.839	±	0.107	bA	3.06	±	0.5	bA	1.75	±	0.08	aA	9.29	±	0.63	aA
	700	WW	1.11	±	0.19	abA	6.63	±	0.88	aA	25.5	±	1.3	aA	1.6	±	0.22	aA	6.5	±	0.59	aA
		MWD	0.924	±	0.151	bA	2.97	±	0.98	abA	10.1	±	1.6	bA	1.51	±	0.14	aB	6.07	±	0.47	aB
		SWD	1.76	±	0.11	aA	2.4	±	0.81	bA	8.82	±	2.55	bA	1.93	±	0.23	aA	8.35	±	0.17	aA
**Icatu**	380	WW	0.83	±	0.07	aB	0.364	±	0.141	aA	25.3	±	2.2	aA	5.33	±	0.95	aA	4.67	±	0.04	bA
		MWD	1.41	±	0.25	aA	1.13	±	0.17	aA	7.89	±	0.98	bB	4.08	±	0.4	aA	6.83	±	0.32	abA
		SWD	1.42	±	0.35	aA	2.23	±	1.16	aA	4.42	±	0.68	bA	6.2	±	0.74	aB	8.81	±	0.33	aA
	700	WW	2.16	±	0.24	aA	2.12	±	0.14	aA	21.8	±	0.8	aA	1.79	±	0.1	cB	4.55	±	0.43	aA
		MWD	1.28	±	0.1	bA	2.82	±	0.32	aA	18	±	3.4	aA	7.89	±	1.1	bA	6.34	±	0.81	aA
		SWD	1.78	±	0.1	abA	2.6	±	0.19	aA	8.33	±	0.22	bA	13.5	±	0.2	aA	6.51	±	0.45	aA
Arabinose				Mannitol				Total Soluble				Starch				* Total Carbohydrates						
1.62	±	0.22	bB	10.7	±	0.7	cA	47.4	±	5.6	cA	33	±	1.3	aB	80.4						
2.33	±	0.24	abA	53.6	±	7.8	bA	76.7	±	8	bA	35.2	±	0.5	aA	112						
2.48	±	0.29	aB	75.8	±	10.3	aA	92	±	11	aA	30	±	0.6	aA	122						
2.64	±	0.18	aA	3.06	±	0.21	cA	45.9	±	1.5	bA	53	±	4.1	aA	98.9						
2.65	±	0.31	aA	17.6	±	3.7	bB	40.4	±	7.6	bB	44	±	1.4	abA	84.4						
3.35	±	0.21	aA	57.5	±	7.7	aB	77.7	±	6.8	aB	38.5	±	1.5	bA	116						
1.71	±	0.2	aA	10.2	±	1.1	cA	48.6	±	3	cA	56.1	±	1.1	aA	105						
2.07	±	0.31	aA	41.2	±	3.9	bA	65.5	±	5.1	bA	47.9	±	1.9	abA	113						
1.92	±	0.27	aA	113	±	6	aA	139	±	5	aA	37.6	±	0.9	bA	177						
0.58	±	0.08	bB	11.8	±	1.2	bA	43.2	±	2.5	bA	52.2	±	2.7	aA	95.4						
1.77	±	0.35	aA	10.6	±	2.4	bB	47.2	±	5.1	bB	51.9	±	1.6	aA	99						
0.795	±	0.145	bB	107	±	5	aA	139	±	7	aA	42	±	0.7	aA	181						

All soluble sugars are expressed in mg g^-1^ DW; starch is expressed in mg glucose equivalents g^-1^ DW). * Total carbohydrates is the sum of the average values of total soluble sugars and starch.

Plants were grown under ambient CO_2_ conditions (380 µL L^-1^, aCO_2_) or elevated CO_2_ (700 µL L^-1^, eCO_2_), and subjected to three water levels: well-watered (WW), moderate water deficit (MWD), and severe water deficit (SWD). For each parameter, different letters after the mean values ± SE (n = 4–6) express significant differences between water availability conditions at the same [CO_2_] (a, b, c) or between the two [CO_2_] under the same water availability condition (A, B), always separately for each genotype.

Drought also strongly depressed sucrose in both genotypes and air [CO_2_], especially under SWD, when values were reduced by 82%–83% (aCO_2_) and 62%–65% (eCO_2_) of their respective WW plants. Still, eCO_2_ clearly mitigated the MWD and SWD impacts in both genotypes, compared with aCO_2_ plants.

Glucose was only marginally increased by drought in both genotypes and air [CO_2_], except in Icatu-MWD and -SWD plants under eCO_2_ where large increases were found. Fructose pools were also slightly affected by drought and air [CO_2_] in both genotypes, with the exception of an increase (89%) in Icatu-SWD plants under aCO_2_, and that SWD plants consistently tended to have somewhat greater contents than WW plants. Arabinose moderately increased upon MWD and/or SWD exposure in both genotypes and [CO_2_]. For each water availability level, eCO_2_ impact on arabinose differed between genotypes, with increases in CL153, and declines in Icatu. Mannitol showed the most remarkable variations under drought among the studied parameters in both genotypes and [CO_2_], usually in a gradual manner as drought severity increased. In SWD plants, mannitol content increased as much as approximately 7- and 18-fold in CL153, and 10- and 8-fold in Icatu, respectively, under aCO_2_ and eCO_2_, with absolute values far greater in Icatu.

eCO_2_ had no impact in WW plants (both genotypes), but attenuated mannitol increase in CL153 (MWD and SWD) and Icatu (MWD).

In general, starch content gradually declined under drought in both genotypes and [CO_2_], except in CL153-aCO_2_ plants that maintained stable values. For each drought level, eCO_2_ moderately increased starch values.

Total soluble and total carbohydrate (containing starch) contents followed a close variation pattern to that of mannitol due to its large absolute values and extent of changes, mostly overcoming those of other sugars. A common species pattern of variation was observed, with SWD constituting the greatest driver for total soluble and total carbohydrates increase in both [CO_2_], but eCO_2_ partly offset the increase in CL153 (MWD and SWD) and Icatu (MWD).

### 3.5 Membrane integrity and dynamics of lipid profile from chloroplasts

**Figure 2:**
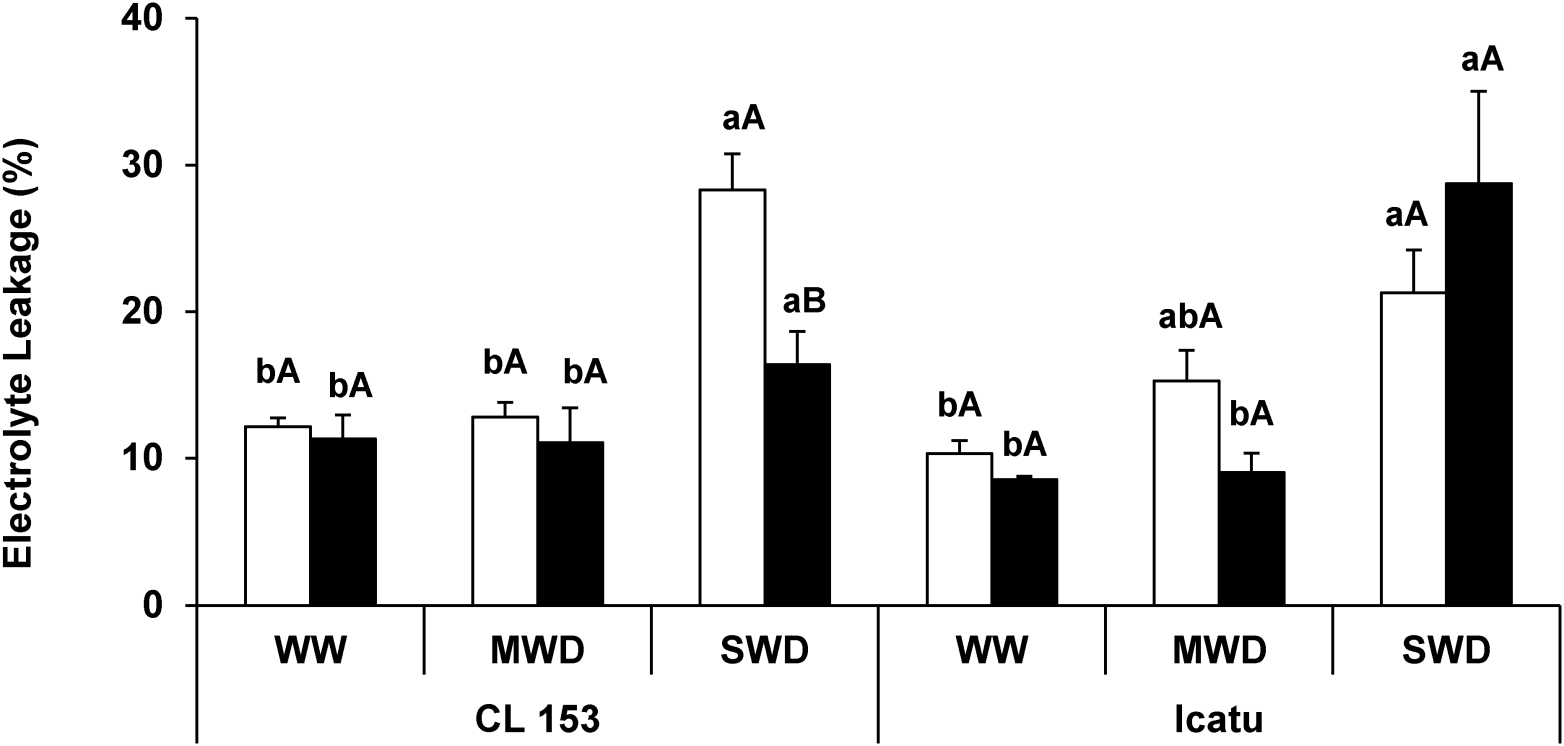
Membrane permeability evaluated as electrolyte leakage (%) in leaf discs from *Coffea canephora* cv. Conilon Clone 153 (CL153) and *C. arabica* cv. Icatu. Plants were grown under ambient CO_2_ conditions (380 µL L^-1^, aCO_2_, white bar) or elevated CO_2_ (700 µL L^-1^, eCO_2_, black bar), and subjected to three water availability levels: well-watered (WW), moderate water deficit (MWD), and severe water deficit (SWD). For each parameter, different letters after the mean values ± SE (n = 5) express significant differences between water availability conditions at the same [CO_2_] **(a, b)** or between the two [CO_2_] under the same water availability condition **(A, B)**, always separately for each genotype.

MWD did not significantly affect cell membrane permeability in both genotypes and [CO_2_] levels, but SWD increased electrolyte leakage in aCO_2_ plants of CL153 (132%) and Icatu (107%) (**Figure 2**). eCO_2_ alone did not alter membrane permeability in WW plants of both genotypes. On the other hand, eCO_2_ reduced electrolyte leakage by approximately 42% in CL153-SWD and Icatu-MWD plants; however, it did not mitigate the impact on Icatu-SWD, always as compared with aCO_2_ plants.

**Figure 3:**
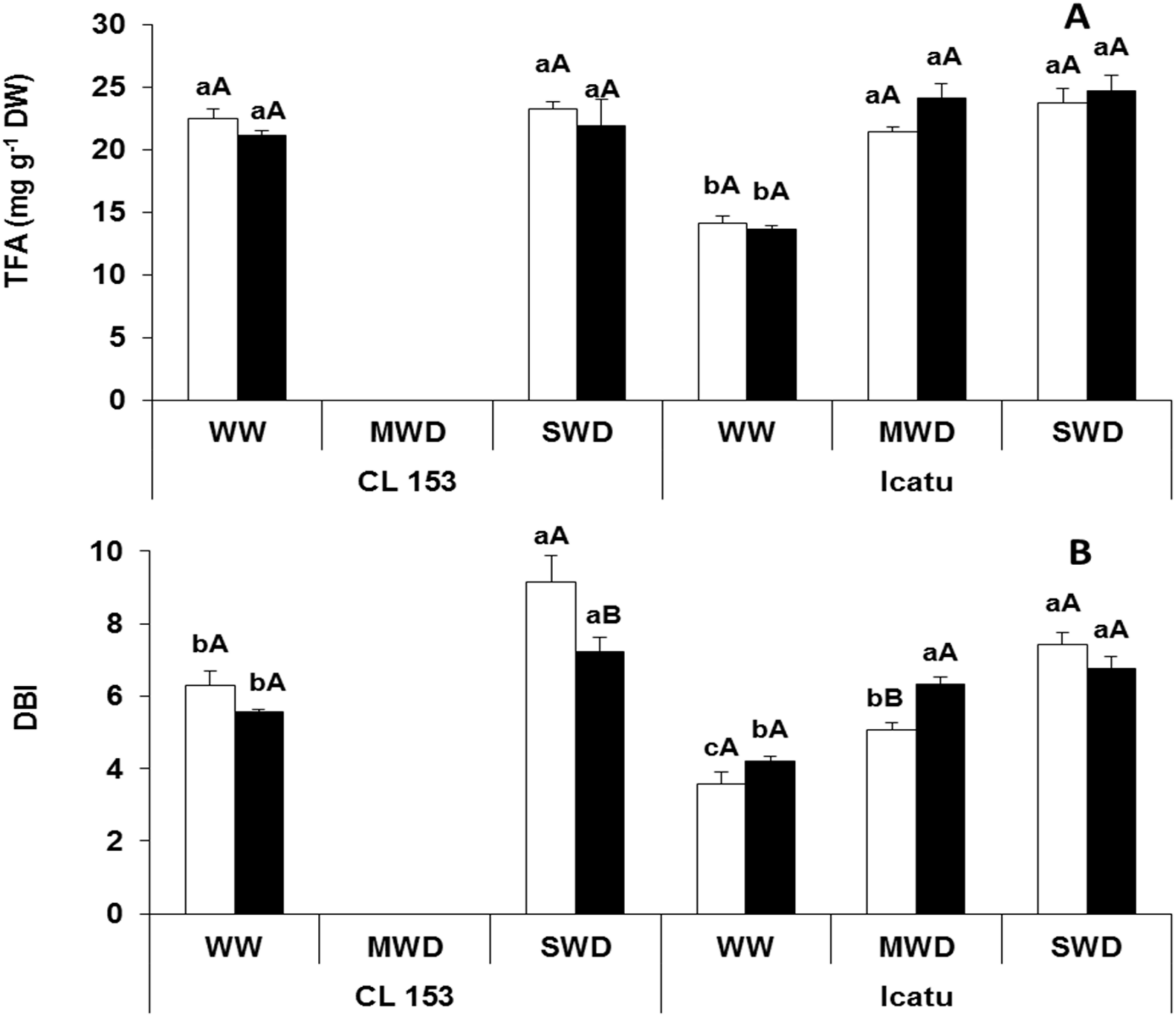
Leaf content of total fatty acids (TFA) **(A)** and the degree of FA unsaturation (as assessed by double bond index, DBI) **(B)**, of the chloroplast membrane lipid matrix, in leaves of *Coffea canephora* cv. Conilon Clone 153 (CL153) and *Coffea arabica* cv. Icatu. Plants were grown under ambient CO_2_ conditions (380 µL L^-1^, aCO_2_, white bar) or elevated CO_2_ (700 µL L^-1^, eCO_2_, black bar), and subjected to three water availability levels: well-watered (WW), moderate water deficit (MWD), and severe water deficit (SWD). The lack of CL153-MWD values is due to sample loss. For each parameter, different letters after the mean values ± SE (n = 3) express significant differences between water availability conditions at the same [CO_2_] **(a–c)** or between the two [CO_2_] under the same water availability condition **(A, B)**, always separately for each genotype.

Regarding the profile of chloroplast membranes, eCO_2_ alone did not alter both total fatty acids (TFA) and the degree of unsaturation (assessed by DBI) in both genotypes (**Figure 3**). As regards SWD alone, TFA content was not quantitatively affected in CL153, but the 45% DBI increase revealed qualitative membrane remodeling toward greater unsaturation based on pre-existing FAs. Icatu plants also showed a gradual qualitative FA change with increasing drought severity, as shown by DBI increases of 42% in MWD and 108% in SWD. Yet, such changes were accompanied by a quantitative increase in TFAs (51% in MWD and 68% in SWD), reflecting a strong *de novo* synthesis of chloroplast membrane FAs only in Icatu.

The eCO_2_ and drought superimposition did not significantly alter TFA content in either genotype, compared with aCO_2_ values. However, under SWD, eCO_2_ moderately attenuated the rise in the degree of FA unsaturation as DBI declined by 21% (CL153) and 9% (Icatu), despite a transient increase of 25% in Icatu-MWD plants.

To improve our understanding of overall FA change, a detailed analysis of the FA profile of chloroplast membranes was performed. Constitutively (WW, aCO_2_), linolenic acid (C18:3), followed by palmitic acid (C16:0) were the most abundant FAs, although CL153 exhibited higher C18:3 and lower C16:0 contents than Icatu (**Table 4**), consistent with the higher DBI value in the former (**Figure 3B**).

**Table 4:** Individual fatty acid (FA) proportions (mol %) in the lipid matrix of chloroplast membranes, including palmitic (C16:0), hexadecenoic (C16:1 *c+t*), stearic (C18:0), oleic (C18:1), linoleic (C18:2), and linolenic (C18:3), extracted from leaves of *Coffea canephora* cv. Conilon Clone 153 (CL153) and *Coffea arabica* cv. Icatu.

Genotype	[CO_2_]	Water treatment	C16:0				C16:1 c+t				C18:0				C18:1				C18:2				C18:3			
	(μL L^−1^)																									
CL153	380	WW	22.4	±	1.2	aA	3.34	±	0.54	aA	7.61	±	0.25	aA	1.76	±	0.02	bB	11.4	±	0.2	aB	53.4	±	1.4	bA
		MWD		–				–				–				–				–				–		
		SWD	18.1	±	1.4	aA	2.80	±	0.08	aA	5.30	±	0.15	bA	2.25	±	0.07	aA	8.41	±	1.30	aA	63.2	±	0.4	aA
	700	WW	26.0	±	0.1	aA	2.13	±	0.23	aA	6.22	±	0.21	aB	2.26	±	0.03	aA	15.0	±	0.4	aA	48.4	±	0.7	bB
		MWD		–				–				–				–				–				–		
		SWD	22.3	±	1.1	aA	3.23	±	0.23	aA	5.16	±	0.03	bA	2.23	±	0.20	aA	8.96	±	0.36	bA	58.1	±	1.0	aB
Icatu	380	WW	33.9	±	1.2	aA	2.42	±	0.29	bB	8.26	±	1.04	abA	2.41	±	0.18	aA	14.9	±	0.9	aA	38.2	±	3.1	cA
		MWD	22.9	±	0.0	bA	3.82	±	0.23	aA	11.38	±	0.02	aA	1.53	±	0.01	bB	12.4	±	0.0	abA	47.9	±	0.1	bB
		SWD	21.8	±	0.9	bA	3.13	±	0.04	abB	5.16	±	0.10	bA	2.11	±	0.02	abA	9.09	±	0.08	bA	58.7	±	0.8	aA
	700	WW	30.1	±	1.3	aA	4.40	±	0.37	aA	7.77	±	0.56	aA	2.11	±	0.17	aA	13.8	±	0.8	aA	41.8	±	0.0	bA
		MWD	20.8	±	0.6	bA	4.05	±	0.20	aA	8.98	±	0.03	aA	2.34	±	0.03	aA	9.74	±	0.14	bA	54.1	±	0.3	aA
		SWD	20.9	±	0.3	bA	4.36	±	0.34	aA	7.51	±	0.64	aA	2.11	±	0.07	aA	10.0	±	0.1	bA	55.1	±	1.2	aA

Plants were grown under ambient CO_2_ conditions (380 µL L^−1^, aCO_2_) or elevated CO_2_ (700 µL L^−1^, eCO_2_), and subjected to three water availability levels: well-watered (WW), moderate water deficit (MWD), and severe water deficit (SWD). Samples of CL153-MWD are not shown as they degraded during processing. For each parameter, different letters after the mean values ± SE (n = 3) express significant differences between water availability conditions at the same [CO_2_] (a, b, c) or between the two [CO_2_] under the same water availability condition (A, B), always separately for each genotype.

Among the changes promoted by the imposed conditions, single SWD prompted major modifications in the FA profile of chloroplast membranes, with enrichment in C18:3 and reductions in C16:0, C18:0, and C18:2 in both genotypes (with MWD usually showing intermediate values), thereby supporting the increase in DBI. Notably, hexadecenoic acid (C16:1*c+t*) increased with drought in aCO_2_ only in Icatu (58% in MWD, and 30% in SWD).

Despite the similar DBI values under both [CO_2_] in WW plants of CL153 and Icatu (**Figure 3B**), eCO_2_ alone prompted a significant decrease in C18:3, while C16:0 and C18:2 tended to increase in CL153. Notably, Icatu-WW plants under eCO_2_ showed stable relative FA abundances (and quantitative TFA, **Figure 3A**), except for C16:1*c+t* that showed a remarkable 82% increase, compared with aCO_2_ (maintained under MWD and SWD superimposition).

During drought, the presence of eCO_2_ prompted FA changes in CL153-SWD plants. Only C18:3 was significantly altered (decreased), but clear increasing trends were observed in C16:0 (23%) and C16:*c+t* (15%), compared with aCO_2_ plants. In contrast, Icatu-SWD plants did not show significant changes in FA composition due to eCO_2_, with the exception of the 39% increase in C16:1*c+t*, compared with aCO_2_. Notably, this represented an 80% increase compared with Icatu-WW plants under aCO_2_.

## 4 Discussion

### 4.1 Impact of water deficit and eCO_2_ on leaf water relations

Under aCO_2_, a gradual decline in Ψ_pd_ occurred as drought severity increased, reaching values close to -3.7 MPa in both genotypes, reflecting extreme water deficit in coffee trees (Pinheiro et al., 2004; Dubberstein et al., 2020). This Ψ_pd_ pattern was paralleled by minor changes in Ψ_π100_, but with a gradual decline in Ψ_p_, showing that cell expansion and growth were already compromised under MWD (Ψ_pd_ close to -2.0 MPa) in aCO_2_ plants due to turgor loss (defined by the absence of positive Ψ_p_) (DaMatta et al., 2003; Almeida et al., 2021), consistent with the increase in unused total soluble carbohydrate (**Table 3**). This aligned with reports showing that zero turgor in *C. arabica* genotypes occurs at RWC ranging from 85% (Catuaí) to 77% (Icatu), and Ψ_pd_ values of approximately −2.2 and −2.5 MPa, respectively, under chilling-induced dehydration (Quartin et al., 2000).

Notably, in either genotype, eCO_2_ alone (WW plants) did not alter water relations parameters (**Table 1**). Yet, eCO_2_ clearly delayed the decline in Ψ_pd_ in MWD plants and retained positive Ψ_p_, especially in Icatu, overall, indicating a less severe stress level (Levitt, 1980). The negative Ψ_p_ values should be interpreted qualitatively as indicators of turgor loss. Therefore, by maintaining positive Ψ_p_ values, the eCO_2_-MWD plants could maintain growth and expansion, and were subjected to a more gradual stress exposure. This allowed a greater response time to the limited water availability, while maintaining a continuous development of vegetative and reproductive structures (Rakočević et al., 2020; 2021), associated with a lower photoinhibition status (PI_Tot_, **Figure 1**), and relevant C-assimilation (Semedo et al., 2021). Lastly, such growth may explain the absence of an increase in total soluble carbohydrates (**Table 3**), preventing photosynthetic downregulation (Ramalho et al., 2013b) and the eCO_2_-induced acceleration of leaf senescence (Yi and Yano, 2025) that could compromise the growth benefits of eCO_2_.

Minor Ψ_π100_ changes were found in MWD and SWD, in both air [CO_2_] and genotypes, compared with WW, but Ψ_π100_ decreased progressively from MWD to SWD, pointing to a moderate osmotic adjustment in Icatu (both air [CO_2_]) and CL153 (aCO_2_), which represents an advantageous plant strategy when facing transient water shortage, e.g., due to irregular rainfall. This Ψ_π100_ decline paralleled the largest increases and absolute contents of total sugars in SWD, largely based on mannitol synthesis (regardless of air [CO_2_]), and glucose (under eCO_2_), and, to a lesser extent, fructose (**Table 3**), but not on sucrose (another sugar with an osmotic role). This aligned with mannitol responsiveness to heat and, especially, drought in *Coffea* spp (Carvalho et al., 2014; Rodrigues et al., 2024), and its accumulation has been associated with tolerance to salt, osmotic, and water stresses, by contributing to osmotic adjustment, cell homeostasis (Stoop et al., 1996; Abebe et al., 2003; Sickler et al., 2007; Saddhe et al., 2021) and protective roles (see below). Finally, glucose pools increased markedly during drought only when combined with eCO_2_ and in Icatu, whereas fructose tended to increase under SWD in both genotypes and air [CO_2_], likely reflecting sucrose catabolism, thus contributing to a modest osmotic adjustment from MWD to SWD (except in CL153-eCO_2_ plants). Overall, our findings showed that eCO_2_ attenuated the decline in both Ψ_pd_ and Ψ_p_ in MWD (greatly in Icatu), preserving the growth ability, and that a moderate osmotic adjustment was possible from MWD to SWD (especially in Icatu) regardless of air [CO_2_], largely associated with mannitol synthesis.

### 4.2 Drought impact on PSII functioning and the eCO_2_ mitigation role

Under WW (or even MWD) conditions, stomatal limitations are the major constraints on *Coffea* spp. photosynthesis due to intrinsically low g_s_ values (DaMatta et al., 2019; Martins et al., 2019). As drought intensifies, mesophyll and/or biochemical constraints become increasingly important, with impacts on the photochemical machinery (Dubberstein et al., 2020; Semedo et al., 2021). The photoinhibition of PSII increases when light energy absorbed by the light harvesting chlorophyll–protein complexes (LHCII) exceeds the capability for photochemical use, with this overexcitation resulting in the overproduction of ROS that causes oxidative damage to cellular components.

Here, PSII photoinhibition was absent under MWD as evidenced by the stability of PI_Chr_ regardless of genotype and air [CO_2_] (**Figure 1A**), in line with the preservation of electron flow activity (Semedo et al., 2021). Under eCO_2_, PI_Dyn_ was also stable (**Figure 1B**), likely due to a greater photochemical use of energy (the best way to avoid overexcitation of the photosynthetic apparatus) (Rodrigues et al., 2016). Under aCO_2_, PI_Dyn_ (and PI_Tot_) increased, particularly in Icatu, but no increase was observed for PI_Chr_, and PI_Dyn_ consistently accounted for the majority of PI_Tot_ in either aCO_2_ or eCO_2_ even in SWD. This reflects the high PSII resilience to drought (even in SWD), supported by the activation of a broad, integrated protective response in both genotypes, especially in Icatu, which included photoprotective pigments (**Table 2**), increased mannitol levels(**Table 3**, see 4.4), greater activity of antioxidative enzymes (e.g., SOD and APX) and cyclic electron flow (CEF) around PSII and/or PSI (Semedo et al., 2021; Marques et al., 2022a), altogether constituting an antioxidant defense system against oxidative stress.

In contrast, CL153 plants showed PSII sensitivity to SWD under aCO_2_, as reflected by the large increase in PI_Chr_ (**Figure 1**), likely due to an imbalance between the damage caused by excess light energy in PSII and the recovery of this process (Murata et al., 2007; Nishiyama and Murata, 2014), when photoprotective mechanisms for energy dissipation and ROS control are insufficient. Partial protection was evidenced by the large increase in PI_Dyn_ (**Figure 1**), and the regulated energy dissipation processes [Y_(NPQ)_] (Semedo et al., 2021). However, despite the absence of an increase in deregulated energy dissipation processes (Y_(NO)_) (which reflect negative non-regulated energy dissipation at PSII, Huang et al., 2011), our findings point to important impacts on the photosynthetic apparatus of CL153-SWD plants under aCO_2_. In fact, the decline in Chl (*a*+*b*) content (**Table 2**), which is associated with environmental stress, is an indicator of plant stress status (Hendry and Price, 1993), and has been related to stress sensitivity to heat (Cui et al., 2006; Lamaoui et al., 2018), drought (Buezo et al., 2019; Ghassemi et al., 2019), and cold (Haldimann, 1999). Moreover, such a decline was accompanied by a reduction in PSs activities and thylakoid electron carriers (Semedo et al., 2021), together with a loss of membrane selectivity (**Figure 2**), indicating altered stability and integrity (Elbasyoni et al., 2017), ultimately affecting photosynthesis and plant growth (Tikkanen and Aro, 2014; Wei et al., 2018). Remarkably, eCO_2_ clearly enhanced CL153-SWD resilience. First, PI_Chr_ returned to the level of WW plants, while PI_Dyn_ maintained high values, thus reflecting the presence of photoprotective mechanisms, such as pigments for thermal energy dissipation (Demmig-Adams and Adams, 1996; Giossi et al., 2020; Dumanović et al., 2021). Second, mitigation, or even cancellation, of the harmful impacts caused by SWD in CL153-aCO_2_ plants was found, regarding membrane leakage (**Figure 2**), Chl (*a*+*b*) values (**Table 2**), PSs activity and thylakoid electron carriers, and triggering the presence of additional protective molecules (e.g., ascorbate) (Semedo et al., 2021; Marques et al., 2022a; Marques et al., 2022b).

The dynamics of the photosynthetic apparatus can also be estimated from the response of the Chl (*a*/*b*) ratio to environmental changes that reflect modifications in the PSs structure (Anderson et al., 2008), with its increase indicating heat sensitivity (Cui et al., 2006). Both PSI and PSII reaction centers are devoid of Chl *b*, which is largely present in LHCII (Chl *a*/*b* ≈ 1.1–1.3; Lichtenthaler and Babani, 2004). Thus, a decline in Chl (*a*/*b*) ratio, similar to that reported in heat-resilient coffee plants (Martins et al., 2016), likely indicates functional readjustments with a preferential LHCII synthesis and its proportion relative to PSII cores (Evans, 1988) rather than selective Chl *a* bleaching (Kitajima and Hogan, 2003). Moreover, Chl *b* is essential for the assembly and photoprotection of PSI, and its deficiency alters the organization of thylakoid proteins, leading to disrupted assembly of PSI complexes, PSI photoinhibition, and reduced photoprotective ability (Mao et al., 2025). In this sense, a greater Chl *b* fraction [reflected by the consistent tendency toward a decline in Chl (*a*/*b*) ratio] may enhance the function and protection of PSI complexes in Icatu (irrespective of air [CO_2_]) and CL153 (under eCO_2_), supporting the claim of a minor disorganization at the level of antenna complex under harsh SWD, consistent with a better photoinhibition status (**Figure 1**, discussed above), and the preservation of the potential performance of photosynthetic structures (PSII, PSI, and thylakoid electron transport).

Nevertheless, remodeling toward greater LHCII abundance increases the potential for light energy capture, increasing the risk of PSs overexcitation when photochemical energy use is limited during drought and heat. Therefore, these findings can also have practical crop management implications, showing the need to reduce the probability of energy (over)load in the photosynthetic machinery, thereby emphasizing the importance of implementing shade management (e.g., through agroforestry systems). This will limit excessive energy input, while improving soil quality, water retention, and biodiversity, balancing plant health, productivity, and environmental conservation, thus constituting a strategy to enhance crop sustainability under a CC context (Koutouleas et al., 2022; DaMatta et al., 2025; Rafique et al., 2025).

Collectively, these findings better explain the role of eCO_2_ in the acquired resilience of CL153 plants to SWD, as also reported for heat in *Coffea* spp (Martins et al., 2016; Rodrigues et al., 2016), and for heat and/or drought in other species (Zinta et al., 2014; Abo Gamar et al., 2019). Still, these changes should be complemented with the activation of several defense responses, including antioxidative molecules, dissipative cycles around PSs (Semedo et al., 2021; Marques et al., 2022b), and greater contributions from protective pigments, sugars, and membrane lipid dynamics, as shown here.

### 4.3 Dynamics of photoprotective pigments

Dissipative pigments are crucial components of the acclimation response of *Coffea* spp. to environmental stresses, such as cold, heat, and high irradiance (Ramalho et al., 2000; Batista-Santos et al., 2011; Vinci et al., 2022). Although the greater presence of photoprotective pigments can be interpreted either as a consequence of a stress already experienced by plants or as a preventive response to avoid severe stress, it is well established that the ability to increase the accumulation/synthesis of such photoprotective pigments is a positive trait that promotes acclimation and is associated with stress resilience. Carotenoids such as Zea, Ant, and lutein act as ^3^Chl^*^, ^1^Chl^*^, and ^1^O_2_ quenchers, and remove epoxy groups from the oxidized double bonds of PUFAs of chloroplast membranes, thus preventing lipid peroxidation in oxygenic photosynthetic organisms (Demmig-Adams and Adams, 1996; Pérez-Bueno and Horton, 2008; Dall'Osto et al., 2012; Demmig-Adams et al., 2022). Here, Tot Car remained invariant to MWD in both genotypes and [CO_2_] (**Table 2**), consistent with the natural resilience of these genotypes to MWD (Semedo et al., 2021) and the absence of major effects on photosynthetic machinery (**Figure 1**). However, the carotenoid pool was reinforced under more severe single drought (SWD) in Icatu, and with eCO_2_ superimposition in both genotypes (compared with SWD-aCO_2_), particularly in Icatu, thus conferring these plants a greater photoprotective ability.

In CL153-SWD plants, only Zea (and DEPS) increased, mostly from the pre-existing (Viol + Ant + Zea) pool, while lutein remained unresponsive to both drought and air [CO_2_]. This contrasted with the strong *de novo* synthesis of most carotenoids [neoxanthin, Zea, accompanied by a greater DEPS, xanthophyll (Viol + Ant + Zea) pool, lutein, and α-carotene] in Icatu-SWD plants (**Table 2**). In addition, the [(Viol+Ant+Zea/Tot Car] ratio gradually increased with drought severity in both genotypes and air [CO_2_], denoting the greater preponderance of the xanthophyll pool. However, only in Icatu-SWD was this associated with a significant increase in Tot Car, reflecting an intrinsic capacity (under aCO_2_) to strengthen photoprotection when the photochemical use of energy is strongly depressed and associated with stomatal closure (Dubberstein et al., 2020). Such a higher carotenoid content, especially Zea, is crucial in protecting membranes against lipoperoxidation (Demmig-Adams et al., 2022), and the Cyt *b_6_/f* complex from ^1^O_2_ (Martins et al., 2016; Ramalho et al., 2018a; Semedo et al., 2021), and aligns with increased PI_Dyn_ (already in MWD for Icatu) (**Figure 1**), and the increase of PsbS protein involved in non-photochemical quenching protective mechanism (Semedo et al., 2021). Still in Icatu-SWD plants, lutein and neoxanthin increases (somewhat greater with eCO_2_ superimposition) can improve acclimation response. Lutein will complement the Zea action through a cycle parallel to that of xanthophylls (Matsubara et al., 2008), removing excess energy in LHCs, mainly through the de-excitation of ^3^Chl* and ^1^O_2_ that are initiators of lipid peroxidation (Adams et al., 2002; Kalituho et al., 2007). In addition, lutein and neoxanthin can better preserve the antennae, since they are integral components of peripheral LHCs, and are crucial for their assembly into trimeric structures, stability, and efficiency (Havaux and Niyogi, 1999; Kalituho et al., 2007; Matsubara et al., 2008). Collectively, these changes enhance LHC*s* stability against excess excitation energy (Havaux and Niyogi, 1999; Niyogi et al., 2005; Ruban, 2016), thus shielding the PS structure and functioning.

Both genotypes showed an increase in the (α/β) carotene ratio, although due to distinct α- and β-carotene variations. Considering the need for a more robust photoprotective capability under SWD, β-carotene [and (α+β) carotene] content surprisingly declined sharply under CL153-SWD plants in either [CO_2_]. In fact, β-carotene is a Zea precursor (Niu et al., 2020), and a quencher of ³Chl and ¹O_2_, crucial for PSs protection and stabilization (De Las Rivas et al., 1993; Faraloni and Torzillo, 2017), and the protection of Cyt *b_6_/f* complex (Zhang et al., 1999). Therefore, such β-carotene depletion reflects a weak point in CL153-SWD plant acclimation, in line with the drought impact on the Cyt *b_6_/f* level (Dubberstein et al., 2020), the higher PI_Chr_ (**Figure 1**), and the membrane leakage under aCO_2_ (**Figure 2**). In contrast, the lower decline in β-carotene in Icatu was fully compensated by an increase in α-carotene, consistent with their role as an antioxidant and lutein precursor (Niu et al., 2020). Finally, most individual carotenoids consistently tended toward greater values in CL153-SWD plants under eCO_2_ than aCO_2_, thus supporting the lower membrane leakage (**Figure 2**) and PI_Chr_, and the higher PI_Dyn_ (**Figure 1**) in these plants.

In short, Icatu exhibited a greater ability to reinforce photoprotective carotenoids when facing SWD, whereas CL153 shows an absence of large increases in these compounds and a β-carotene loss, thus increasing its vulnerability. eCO_2_ amplified the protective response of carotenoids to SWD in both genotypes, thus supporting a better photosynthetic performance, and the impact of mitigation in CL153 compared with aCO_2_.

### 4.4 Prospective protection of specific sugars

Mannitol is the most abundant polyol in plants (Loescher, 1987), with a known osmotic role without the negative effects on photosynthesis caused by the accumulation of other sugars, such as glucose, fructose, and sucrose (Lee et al., 2008; Keunen et al., 2013). In addition, mannitol can reduce oxidative damage in chloroplasts through the hydroxyl (^●^OH) and superoxide (O_2_^●-^) radical scavenging (Shen et al., 1997), complementing other mechanisms such as photoprotective pigments (**Table 2**) and antioxidative molecules (Semedo et al., 2021; Marques et al., 2022a; Marques et al., 2022b), even though its concentrations are insufficient to account for a meaningful osmotic adjustment (Abebe et al., 2003). This interpretation is consistent with our results, where a marked increase in mannitol pools (**Table 3**) corresponded only to a modest osmotic adjustment from MWD to SWD conditions (**Table 1**), but was accompanied by the absence of PI_Chr_ aggravation (**Figure 1**) and the preservation of PSII and photosynthetic apparatus functionality in Icatu-SWD plants (Semedo et al., 2021). Notably, mannitol accumulation was attenuated by eCO_2_ under MWD (both genotypes), likely because the photochemical use of energy through C-assimilation remained higher than in aCO_2_ plants, and other protective systems were already in place (Marques et al., 2022b). In fact, such defenses reached near-maximal levels under MWD, thus requiring additional reinforcement under the harsher SWD conditions. This aligned with the greatest increase and absolute contents of mannitol in SWD plants of both genotypes (particularly in Icatu), as reported under severe heat (42 °C) (Rodrigues et al., 2024). This increase in mannitol was partly supported by sucrose (Saddhe et al., 2021), and starch breakdown (especially under SWD), but the scale of its accumulation far exceeded the combined declines in sucrose and starch. This points a robust *de novo* synthesis from other C sources, particularly evident in Icatu, and to the high plasticity and coordinated regulation of protective and sugar-metabolic pathways.

Changes in other protective sugars were quite limited, such as those of the trisaccharide raffinose, known for its pivotal role as a compatible solute in desiccation and acquired stress tolerance, stress signaling, and antioxidant defense (Lahuta and Górecki, 2011; ElSayed et al., 2014; Yan et al., 2022), stabilizing thylakoid membrane, and sustaining electron transport (Santarius, 1973), including under heat in *Coffea* spp. (Santos et al., 2011; Dubberstein et al., 2020).

Overall, drought increased total soluble and total carbohydrates, largely due to the increase in mannitol, and despite declines in sucrose and starch. Under MWD, these changes in carbohydrates were usually offset by eCO_2_, but under SWD, the drought response predominated, overcoming the eCO_2_ effect on carbohydrates.

### 4.5 Membrane integrity and lipid dynamics: a key trait of drought resilience

Cell membranes are primary targets of stress and early indicators of stress sensitivity (Mano, 2002), but are also crucial sites for perceiving environmental stimuli and triggering acclimation responses (Osakabe et al., 2014; Hou et al., 2016; Niu and Xiang, 2018). Membrane permeability, a proxy for stress tolerance, namely, in *Coffea* spp (Peloso et al., 2017; Ramalho et al., 2018a), showed only minor impacts caused by MWD, irrespective of genotype and air [CO_2_] (**Figure 2**), consistent with the preservation of photosynthetic performance (Semedo et al., 2021; Marques et al., 2022b). Under single SWD, the greater leakage in both genotypes could point to altered membrane stability and integrity (Elbasyoni et al., 2017), associated with the loss of cell compartmentalization and metabolic dysfunction (Scotti-Campos et al., 2019). Still, lipoperoxidation did not increase in either genotype (Rodrigues et al., 2024), and increased PI_Chr_, with impacts on the photosynthetic apparatus (e.g., electron carriers), only in CL153 but not in Icatu, where energy-dissipative and photoprotective mechanisms (**Table 2**), a strong antioxidative response, and the presence of CEF associated with both PSs were observed (Semedo et al., 2021; Marques et al., 2022b; Rodrigues et al., 2024). Furthermore, eCO_2_ reverted most of the impacts on CL153-SWD plants, including those on PI_Chr_, membrane permeability (**Figures 1**, **2**), and thylakoid electron transport (Semedo et al., 2021), which is a membrane-based event directly affected by thylakoid membrane dysfunction (Nouri et al., 2015). This was concomitant with an enhanced expression of water- and stress-responsive genes and reinforced antioxidative activity (e.g., Cu, Zn-SOD and APX) (Semedo et al., 2021; Marques et al., 2022b; 2023), similar to the mitigation of heat impacts by eCO_2_ (Martins et al., 2016; Rodrigues et al., 2016), altogether confirming the role of eCO_2_ in the preservation of membranes and the events directly related to them under harsh SWD conditions in both genotypes.

Notably, membrane permeability increased under eCO_2_ in Icatu-SWD, without being associated with impacts on Chl (**Table 2**), photosynthetic components, or Y_(NO)_ (Semedo et al., 2021). Instead, it likely resulted from membrane remodeling rather than injury, as reported under heat (Scotti-Campos et al., 2019).

Modulation of the lipid profile of chloroplast membranes is decisive for preserving metabolic functions, and a higher degree of unsaturation enhances chloroplast membrane fluidity and sustains C-assimilation (Gombos and Murata, 1998; Routaboul et al., 2000), which is crucial to drought acclimation (Xin and Browse, 2000; Perlikowski et al., 2016; Zhang et al., 2018; Liu et al., 2019). In fact, both genotypes increased their unsaturation (DBI) in response to SWD (**Figure 3**), supported by the preferential synthesis of C18:3, and declines in C16:0, C18:0m and C18:2 (**Table 4**), in line with the association of membrane enrichment in C18:3 with drought tolerance in grapevine (Toumi et al., 2008), tea (Chang et al., 2024), and maize (Yin et al., 2024), as well as tolerance to multi-stresses (cold, drought, and salt) in tobacco (Shi et al., 2018). Such unsaturation was slightly attenuated by eCO_2_, mainly due to a smaller increase in C18:3 than in aCO_2_, which is consistent with the absence of eCO_2_ impact on FA composition in *A. thaliana* (Zinta et al., 2018). Still, while changes in unsaturation in CL153 plants relied on pre-existing predominant FAs (TFA content remained unaltered), those in Icatu were also supported by quantitative (*de novo*) FA synthesis. This aligned with the distinct expression of genes related to lipid metabolism in these genotypes (Fernandes et al., 2021), revealing the greater lipid metabolic flexibility of Icatu, which is a key trait for thermal stress tolerance in *Coffea* spp (Partelli et al., 2011; Scotti-Campos et al., 2019). Interestingly, this FA could also act through the C18:3-induced integrated regulation of membrane, Ca^2+^, ROS, and stress-responsive genes (Shi et al., 2018).

Photosynthetic performance has been shown to be closely associated with increased FA unsaturation, which is essential for maintaining lipid acyl movement in thylakoid membranes (Harwood, 1998; Siegenthaler and Tremolieres, 1998). This directly supports electron support in PSI and PSII, both of which are membrane-dependent processes. Moreover, a high degree of FA unsaturation is essential for integrating newly synthesized D1 protein during the PSII repair cycle, thereby sustaining PSII functionality under stress (Kern et al., 2009). Therefore, the larger changes observed in the FA profile and degree of unsaturation in Icatu are mechanistically consistent with the greater preservation of photosynthetic machinery components (e.g., thylakoid electron carriers) and potential under SWD, irrespective of air [CO_2_] (Dubberstein et al., 2020). Still, the enrichment of membranes in polyunsaturated FAs can enhance their susceptibility to lipoperoxidation, since double bonds are preferential targets of hydrolytic enzymes, peroxidases, ROS, and free radicals (Öquist, 1982; Girotti, 1990; Ferrari-Iliou et al., 1993). Consequently, to preserve chloroplast membranes and sustain photosynthetic function, a concomitant enhancement of defense mechanisms must be activated. This was the case in Icatu-SWD plants that triggered multiple response mechanisms, particularly for oxidative stress control. This encompassed the accumulation of protective sugars (e.g., mannitol) and photoprotective pigments (e.g., Zea and lutein) reported in the present study, together with stress-related proteins (HSP70, chaperonins), membrane-stabilizing protectants with antioxidative properties (e.g., aquaporins and dehydrins), and enhanced activity of antioxidant enzymes (Semedo et al., 2021; Marques et al., 2022b; 2023; Rodrigues et al., 2024; Ramalho et al., 2025). In other species, such coordinated responses collectively contributed to the reduction of lipid peroxidation, preserving membrane stabilization and integrity (Patel and Mishra, 2021; Szlachtowska and Rurek, 2023). Thus, despite the increase in DBI, CL153-SWD plants showed a more limited activation of complementary defenses under aCO_2_ (than Icatu-SWD plants), but eCO_2_ elicited a more robust, multi-layered response that mitigated/cancelled the SWD impacts observed under aCO_2_ (Semedo et al., 2021; Marques et al., 2022b; Rodrigues et al., 2024).

Interestingly, only Icatu exhibited an increase in C16:1*c+t* levels from WW to SWD under aCO_2_, with even greater values under eCO_2_. This larger C16:1*c+t* presence resulted from greater relative proportion (**Table 4**) and from the increased TFA abundance (**Figure 3**). The C16:1*t* is a specific FA from thylakoid phosphatidylglycerol (PG) (Öquist, 1982; Siegenthaler, 1998), the sole phosphoglycerolipid synthesized in the chloroplast envelope from phosphatidic acid (Boudière et al., 2014). Therefore, an increase in C16:1*t* also reflects a greater PG abundance, both being critical for the structural interaction with proteins and for the supramolecular organization of thylakoid membranes, particularly involving LCHII proteins and pigments, but also of PSI, thereby stabilizing PS complexes and facilitating the transition between states 1 and 2 and an effective non-cyclic electron transfer (Siegenthaler and Tremolieres, 1998; Vijayan et al., 1998; Dubertret et al., 2002; Yang et al., 2005; Boudière et al., 2014). Moreover, C16:1*t* plays a role in the replacement of damaged D1 protein of PSII (Horváth et al., 1987; Harwood, 1998; Gray et al., 2005), facilitating faster recovery from the PSII photoinhibited state (Gombos and Murata, 1998; Siegenthaler and Tremolieres, 1998). Thus, a greater abundance of C16:1*c+t* in Icatu-SWD plants mechanistically aligns with a photosynthetic apparatus resilience to drought, as shown by stable PI_Chr_ (**Figure 1**), the lack of negative impacts on thylakoid components in either air [CO_2_] (Semedo et al., 2021), and their superior recovery from heat stress (Dubberstein et al., 2020).

Overall, together with additional protective defenses, the flexible reprogramming of the chloroplast lipid matrix, *via* both quantitative (*de novo* synthesis) and qualitative (unsaturation shifts) adjustments, plus C16:1*c*+*t* modulation, emerged as key responses to drought in Icatu, while eCO_2_ enhanced these responses in CL153, reversing SWD impacts.

## 5 Conclusions

Drought intensification to the extreme SWD level (Ψ_pd_ ≤ -3.7 MPa) triggered turgor loss, but eCO_2_ attenuated the decline in Ψ_pd_ and preserved cell expansion ability (Ψ_p_ > 0) under MWD, mainly in Icatu. Although Ψ_π100_ remained largely unchanged across [CO_2_] and genotypes relative to WW plants, the decline in Ψ_π100_ from MWD to SWD in Icatu (both air [CO_2_]) and in CL153 (aCO_2_) indicates a moderate osmotic adjustment, likely associated with maximum mannitol (and glucose under eCO_2_) abundance.

The resilience of PSII under MWD, as indicated by the invariant PI_Chr_ in both genotypes, was primarily sustained by enhanced energy use under eCO_2_ and by the activation of photoprotective mechanisms under aCO_2_ (PI_Dyn_ rise). Under SWD, PI_Dyn_ remained the dominant component across all conditions, but Icatu maintained stable Chl (*a*+*b*) and PI_Chr_ values while exhibiting an enhanced PI_Dyn_ indicating greater resilience supported by a coordinated suite of responses. These included greater accumulation of mannitol, *de novo* synthesis of photoprotective carotenoids [neoxanthin, zeaxanthin, (Viol+Ant+Zea) pool, and lutein], and adjustments in PSs [a decline in Chl (*a*/*b*) ratio]. Collectively, these traits contributed to the preservation of photosynthetic functionality, thereby underpinning acclimation to SWD. In contrast, despite an increase in PI_Dyn_ under aCO_2_, PI_Chr_ increased in CL153-SWD plants, along with a decline in Chl (*a*+*b*), greater membrane leakage, and PSII impairments, thus indicating incomplete photoprotection. Notably, eCO_2_ largely reduced these impacts, particularly by enhancing the presence of photoprotective pigments, thereby playing a key role in the resilience to drought in CL153-SWD plants.

Both genotypes adjusted their lipid matrix in response to drought, exhibiting qualitative changes (increased membrane unsaturation). However, only Icatu displayed strong quantitative adjustments, associated with declines in C16:0 and C18:2 and a pronounced *de novo* synthesis of C18:3 and C16:1*c+t* (the latter being larger at eCO_2_). Because unsaturated FAs are more prone to oxidative damage, such lipid adjustments necessarily require the concomitant activation of antioxidative defenses, consistent with the accumulation of photoprotective pigments and compatible solutes such asmannitol.

Together, our findings highlight the intrinsic resilience of both genotypes under MWD, the superior tolerance of Icatu to SWD (even under aCO_2_), and the pivotal role of eCO_2_ in enhancing coffee acclimation to drought by mitigating or reversing most of the impacts observed under aCO_2_ in CL153.

Collectively, these responses are likely critical and reflect key traits underpinning drought resilience and the long-term sustainability of *Coffea* spp. under future climate-change scenarios, and should receive particular attention as early stress biomarkers for the selection and breeding of new climate-resilient coffee cultivars.

## Data Availability

The original contributions presented in the study are included in the article/supplementary material. Further inquiries can be directed to the corresponding authors.
